# Atypical induction of HIF-1α expression by pericellular Notch1 signaling suffices for the malignancy of glioblastoma multiforme cells

**DOI:** 10.1007/s00018-022-04529-2

**Published:** 2022-10-02

**Authors:** Jungwhoi Lee, Eunsoo Kim, Kyuha Chong, Seung-Wook Ryu, Chungyeul Kim, Kyungsun Choi, Jae-Hoon Kim, Chulhee Choi

**Affiliations:** 1 Department of Applied Life Science, Sustainable Agriculture Research Institute (SARI), Jeju National University, 102 Jejudaehak-ro, Jeju, Jeju-do 63243 Republic of Korea; 2 ILIAS Biologics Inc, 40-20, Techno 6-ro, Yuseong-gu, Daejeon, 34014 Republic of Korea; 3 Department of Neurosurgery, Korea University Guro Hospital, Korea University Medicine, Korea University College of Medicine, 148 Gurodong-ro, Guro-gu, Seoul, 08308 Republic of Korea; 4 Laboratory of Photo-Theranosis and Bioinformatics for Tumors, Department of Neurosurgery, Samsung Medical Center, 81 Irwon-Ro, Gangnam-gu, Seoul, 06351 Republic of Korea; 5 Department of Neurosurgery, Samsung Medical Center, Sungkyunkwan University School of Medicine, 81 Irwon-Ro, Gangnam-gu, Seoul, 06351 Republic of Korea; 6 Department of Pathology, Korea University Guro Hospital, Korea University Medicine, Korea University College of Medicine, 148 Gurodong-ro, Guro-gu, Seoul, 08308 Republic of Korea; 7 Department of Bio and Brain Engineering, KAIST, 291 Daehak-ro, Yuseong-gu, Daejeon, 34141 Republic of Korea

**Keywords:** Abnormal HIF-1α, Notch1, GBM, Juxtacrine signaling, Chemoresistance

## Abstract

Contact-based pericellular interactions play important roles in cancer progression via juxtacrine signaling pathways. The present study revealed that hypoxia-inducible factor-1α (HIF-1α), induced even in non-hypoxic conditions by cell-to-cell contact, was a critical cue responsible for the malignant characteristics of glioblastoma multiforme (GBM) cells through Notch1 signaling. Densely cultured GBM cells showed enhanced viability and resistance to temozolomide (TMZ) compared to GBM cells at a low density. Ablating Notch1 signaling by a γ-secretase inhibitor or siRNA transfection resensitized resistant GBM cells to TMZ treatment and decreased their viability under dense culture conditions. The expression of HIF-1α was significantly elevated in highly dense GBM cells even under non-hypoxic conditions. Atypical HIF-1α expression was associated with the Notch1 signaling pathway in both GBM and glioblastoma stem cells (GSC). Proteasomal degradation of HIF-1α was prevented by binding with Notch1 intracellular domain (NICD), which translocated to the nuclei of GBM cells. Silencing Notch1 signaling using a doxycycline-inducible Notch1 RNA-interfering system or treatment with chetomin, a HIF pathway inhibitor, retarded tumor development with a significant anti-cancer effect in a murine U251-xenograft model. Using GBM patient tissue microarray analysis, a significant increase in HIF-1α expression was identified in the group with Notch1 expression compared to the group without Notch1 expression among those with positive HIF-1α expression. Collectively, these findings highlight the critical role of cell-to-cell contact-dependent signaling in GBM progression. They provide a rationale for targeting HIF-1α signaling even in a non-hypoxic microenvironment.

**Supplementary Information:**

The online version contains supplementary material available at 10.1007/s00018-022-04529-2.

## Introduction

Glioblastoma multiforme (GBM) is one of the most fatal primary brain tumors with extremely high recurrence rates due to its unique and complex microenvironment within the brain [1]. Targeting brain tumors is a considerable therapeutic challenge and patients with GBM survive only 1–2 years after diagnosis [2]. Temozolomide (TMZ) is the first-line chemotherapeutic DNA-alkylating drug for the treatment of GBM [3]. Although TMZ is one of the anti-cancer drugs with the highest bioavailability and efficiency for the treatment of GBM, the drug response rate is limited to about 40% and GBM patients eventually die due to cancer recurrence and progression [4]. Various signaling pathways including RTK/pI3K/AKT [5, 6], mTOR [7], p53 [8], RAF [9], and MEK/MAPK [10] are adversely regulated in the microenvironment of brain tumors, leading to the acquisition of a growth advantage, resistance to apoptosis, and migratory or metastatic properties. Although considerable effort has been invested in the attempt to develop therapeutic reagents with innovative strategies that target GBM, the overall survival of GBM patients remains unchanged.

Tumor cells flourish within a complex environment characterized by hypoxia which plays a critical role in tumor development [11, 12]. Hypoxic-induced signaling can select for tumor cells so that they effectively adapt to their adverse microenvironment and promote tumor progression by inducing angiogenesis, chemoresistance, evasion of the immune system, and cancer stemness. These mechanisms utilize hypoxia-inducible factor-1α (HIF-1α), which is continuously expressed and specifically preserved in a low oxygen environment and is associated with von Hippel-Lindau (VHL) disease and prolyl hydroxylase (PHD) [13–15]. A broad understanding of tumor cells’ adaptive signaling pathway using HIF-1α/β might be critical for improving therapeutic strategies against malignant tumors.

Along with various extracellular cues such as chemical factors, autocrine/paracrine growth factors, and oxygen concentrations, juxtacrine signaling is also a key signaling pathway between cells in direct contact. It regulates several pathways including cadherins, integrin, and Notch1 signaling pathways [16, 17]. Additionally, Notch1 signaling plays a role in the features of cancer stem cells [18–20]. Proteolytic γ-secretase can cleave intact Notch1 to Notch1 intracellular domain (NICD) in the cytosol, which in turn, can translocate to the nucleus to function as a transcription factor [21].

The overall effects of HIF-1α under hypoxic conditions have been extensively studied and have revealed their involvement in the conventional oncogenic responses of various malignant tumors. The objective of this study was to determine whether cell-to-cell contact could evoke the malignant progression of GBM by preserving functional HIF-1α in consort with the Notch1 signaling pathway, even in a non-hypoxic environment.

## Materials and methods

### Overview

To investigate the correlation and its significance between HIF-1α, one of the important cell signals involved in GBM malignancy, and Notch1, which is involved in cell-to-cell interactions, even in non-hypoxic conditions, experimental verifications such as in vitro, in vivo, and patient sample analyses were performed. For the experimental preparation, a number of GBM cell lines were prepared, and for comparison with these, fibrosarcoma and pancreatic cancer cell lines, which are other highly malignant types of cancers, were prepared. To understand the role of Notch1 and its relationship with HIF-1α in GBM, an experimental environment was created in which the degree of cell-to-cell contact was altered. For the phenotypical evaluation of changes in proliferation and drug susceptibility in an environment in which cell-to-cell contact was controlled, the cell viability assay, production of Notch1-specific shRNA stable cell lines, and in vivo experiments were performed. And to verify the relationship between Notch1 and HIF-1a cell signals and evaluate the effect of regulating these signals on GMB malignancy, experiments such as immunoblot assays, immunoprecipitation assays, HIF-1α activity reporter assays, next-generation sequencing, and siRNA transfection were planned and performed. To confirm clinical significance, a tissue microarray study of GBM patients was designed and the correlation between Notch1 and HIF-1α expression was confirmed using immunohistochemical methods and the results were analyzed by a pathologist blinded to the sample identities.

### Cell culture

Human GBM cell lines (U251-MG, LN215-MG, CRT-MG, and LN215-MG) and fibrosarcoma HT-1080 cells were obtained from the American Type Culture Collection (Manassas, VA, USA). CRT-MG and LN215-MG cells were kindly provided by Professor Etty N. Benvenite (University of Alabama, Birmingham, USA). The human pancreatic cancer cell line (Panc-1) was purchased from the Korean Cell Line Bank (Seoul, Korea). U251-MG, LN215-MG, and Panc-1 cells were maintained in Dulbecco’s modified Eagle’s medium (DMEM, Welgene, Seoul, Republic of Korea) supplemented with 10% fetal bovine serum (FBS, Atlas Biologicals, CO, USA) at 37 °C with an atmosphere of 5% CO_2_. U373-MG and CRT-MG cells were maintained in Roswell Park Memorial Institute 1640 medium (RPMI 1640) with L-glutamine (Welgene) supplemented with 10% FBS (Atlas Biologicals, Fort Collins, CO, USA). Glioblastoma stem cells were cultured in DMEM: nutrient mixture F-12 (DMEM/F12) medium (Welgene) supplemented with B27 (Thermo Fisher Scientific, Seoul, Republic of Korea), 0.02 mg/L of basic fibroblast growth factor (bFGF), and epidermal growth factor (EGF) (Sigma-Aldrich, St. Louis, MO, USA). For all media, 1 × 10^5^ unit/L penicillin and 100 mg/L streptomycin (Invitrogen, Carlsbad, CA, USA) were supplemented. Under standard culture conditions, the cells retained their typical original morphology throughout the periods required to perform the experiments reported in this study.

### Regulation of culture density

Low- or high-density culture conditions were achieved by seeding the same number of cells into culture dishes of different sizes. For low density (Low), 5 × 10^5^ cells were seeded into 100 mm culture dishes (Corning Costar, Lowell, MA, USA, Corning® 100 mm TC-treated culture dish, #430167) containing 7 ml culture medium, while the same number of cells was seeded into 60 mm culture dishes (Corning Costar, Corning® 60 mm TC-treated culture dish, #430167) containing 7 ml culture medium to achieve high density (High). Both groups of cells were cultured identically excepting culture dishes sizes.

### Cell viability assay

Cell viability was determined using a WST-1 assay (Abcam, Cambridge, UK) as described previously [22]. Absorbance at 450 nm was measured using a microplate reader (Bio-Rad, Richmond, CA, USA) after incubation at 37 °C for 10 min.

### Reagents

Temozolomide (TMZ), DAPT (γ-secretase), and 18β-glycyrrhetinic acid (β-GA) were purchased from Sigma-Aldrich. RGDfV peptide (RGDfV) and A205804 were purchased from Santa Cruz Biotechnology (CA, USA). Recombinant soluble Dll4 was obtained from R&D Systems (Minneapolis, MN, USA). MG132 was purchased from Calbiochem (Darmstadt, Germany). Antibodies against Notch1, hydroxyl-HIF-1α, von Hippel–Lindau (VHL), poly (ADP-ribose) polymerase (PARP), and cyclin D1 were purchased from Cell Signaling Technology (Beverly, MA, USA). HIF-1α and prolyl hydroxylase1 (PHD1) were obtained from Abcam. Glyceraldehyde 3-phosphate dehydrogenase (GAPDH) and p65 were purchased from Santa Cruz Biotechnology (Dallas, TX, USA).

### Immunoblot assay

To evaluate the endogenous levels of HIF-1α in various cancer cells at different cell densities, Western blotting was performed as described previously [23]. To detect the attached antibodies, the blots were developed using an enhanced chemiluminescence buffer (AbFrontier, Seoul, Republic of Korea). Band intensities were measured by densitometry using ImageJ software (National Institutes of Health, Bethesda, MD, USA).

### Immunoprecipitation assay

U251-MG and HT-1080 cells were cultured at high or low density. After two days of culture, cell lysates were extracted with lysis buffer (1% NP-40, 20 mM pH 7.5 Tris–HCl, 137 mM NaCl, 2 mM pH 8.0 EDTA, and a protease inhibitor and phosphatase inhibitor cocktail). The cell lysates were incubated with anti-Notch1, anti-HIF-1α, or normal IgG antibodies. Protein G agarose (Amersham Bioscience, Little Chalfont, UK) was then added. After centrifuging, the bound proteins were separated by sodium dodecyl sulfate–polyacrylamide gel electrophoresis (SDS-PAGE) and analyzed by immunoblot assays.

### Cell fractionation

The nuclear/cytosolic fractionation of cells cultured at different cell densities was performed with a Fractionation Kit (Bio Vision, Mountain View, CA, USA) according to the manufacturer’s instructions. The extracted proteins were separated by SDS-PAGE and the cytoplasmic fractions were probed with GAPDH while the nuclear fractions were probed with an PARP-specific antibody.

### HIF-1α activity reporter assay

The cells were transfected with a reporter plasmid containing HIF-1α-responsive elements and a Renilla luciferase gene. After 48 h of transfection, luciferase activity was measured with a luciferase assay system (Promega, Madison, WI, USA). Luminescence intensity was determined with a Wallac multilabel counter (PerkinElmer Wallac, Gaithersburg, MD, USA).

### siRNA transfection

The transfection of small interfering RNAs (siRNA) was performed using Effectene reagent (Qiagen, Hilden, Germany) as described previously[24]. siRNAs against Notch1, HIF-1α, and PSEN1 were purchased from Bioneer (#4851-1, #3091-1, and #5663-1 Daejeon, Republic of Korea) and Santa Cruz Biotechnology (sc-36095, sc-35561, and sc-36312). Scrambled control (sc-37007) was purchased from Santa Cruz Biotechnology. The transient transfection of each siRNA was performed using Lipofectamine 2000™ (Invitrogen) according to the manufacturer’s protocol. The efficiency of siRNA transfection was evaluated by immunoblot analysis.

### Identification of overexpressed genes by next-generation sequencing (NGS)

Cells seeded into 60 mm or 100 mm culture dishes were incubated for 24 h. The total RNA of the high- or low-density cultured cells was extracted using an RNeasy Mini Kit (Qiagen, Valencia, CA, USA) according to the manufacturer’s protocol. Transcriptome sequencing was performed by Macrogen (Seoul, Republic of Korea). Genes overexpressed in the high-density cultured cells (OEG-high) compared to the low-density cultured cells were identified. A target gene list of transcription factors was obtained from the Harmonizome database, and the target genes of a transcription factor ‘x’ were designated as TF(x)_targets. For each transcription factor, the target genes that overlapped with OEG-high were determined and termed TF(x)-high. The relationship between OEG-high and TF(x)-high was analyzed using Fisher’s exact test. The transcription factors with *p*-values below 0.001 were selected, and the ratio of TF(x)-high to TF(x)-targets was calculated (TF(x)_ratio).

### GBM patient tissue microarray (TMA) preparation and interpretation

Patient tissue samples from 19 adults aged 18–75 years who were diagnosed with GBM, which were donated and preserved in paraffin blocks after surgery and pathologic diagnosis, were used. TMA slides consisting of a total of 57 tissue samples from the patients were prepared. Tissue cores 2 mm in diameter were carefully transferred to recipient paraffin blocks. The filled recipient blocks were embedded in paraffin and 3-µm-thick sections were cut and mounted on slides. The TMA slides were dewaxed by heating at 55 °C for 30 min followed by three 5-min washes with xylene and rehydration by 5-min washes in a graded ethanol series of 100, 95, and 80% (v/v) and finally, pure distilled water. Antigen retrieval was performed by heating the sections at 95 °C for 30 min in 10 mM sodium citrate (pH 6.0). Endogenous peroxidase activity was blocked by incubating in 3% (w/v) hydrogen peroxide for 30 min. The background reactivity was removed using a universal blocking serum (Dako Diagnostics, Glostrup, Denmark) for 30 min at room temperature. The slides were incubated for 1 h with antibodies specific to HIF-1α (ab16066, 1:100; Abcam) and Notch1 (ab52627, 1:100; Abcam), and then for 30 min with a biotin-labeled secondary antibody. Streptavidin-peroxidase (Dako Diagnostics) was applied and developed. After slight counterstaining with hematoxylin, the slides were dehydrated and mounted under coverslips for microscopy. After immunostaining, a pathologist analyzed the level and presence of HIF-1α and Notch1 through a blinded review. Staining intensity was scored from none or 1 + (very weakly positive) to 4 + (very strongly positive).

### Generation of Notch1-specific shRNA stable cell lines

For the stable suppression of Notch1 expression by a short hairpin (sh)-activated gene silencing vector system, plasmids expressing sh-RNAs were constructed by synthesizing cDNA oligonucleotides bearing the target sequence and *Xho*I and *Hind*III linkers, and then ligated into the *Xho*I and *Hind*III sites of a pSingle-tTs-shRNA vector (tTs-VEGF shRNA, Clontech). The target sequences for Notch1 were 5′-GAACGGGGCUAACAAAGAUUU-3′. They were synthesized accordingly. One day after transfection with tTs-Notch1 shRNA constructs, U251-MG cells were grown in Dulbecco’s complete medium containing 1 g/L G418 for 3 days followed by an additional 6 days of culture in Dulbecco’s complete medium containing 400 mg/L G418 for selecting stable transfectants. The cells were then subjected to Western blot analysis for Notch1 expression.

### Xenograft tumor model

Balb/c nude mice were obtained from Orient (Seongnam, Korea) at 5 – 6 weeks of age. All mice were housed and handled in accordance with the Animal Research Committee’s Guidelines at KAIST (Daedeok Science Town, Daejeon, Republic of Korea). When the tumors reached an average size of approximately 200 mm^3^, 200 mg/L of doxycycline in water was provided to the mice or different dosages of chetomin were administrated by intraperitoneal injection. Tumor volume was calculated using the following formula: *V* = 0.523 *LW*^2^ (L = length, W = width). Body weights were recorded regularly.

### Ethics approval and consent to participate

The patient sample study complied with guidelines and protocols approved by the Korea University Guro Hospital Institutional Review Board (IRB No. 2017GR0330).

### Statistical analysis

All experimental data are presented as the mean ± standard deviation (SD). Student’s *t* test and Mann–Whitney test were used to determine the levels of significance for comparison between two independent samples. The significance of differences between sample groups was evaluated by one-way analysis of variance (ANOVA) with Tukey’s post hoc test for significant main effects.

## Results

### High cell-to-cell contact induces pro-tumorigenic features in various glioblastoma multiforme (GBM) cells

**Fig. 1:**
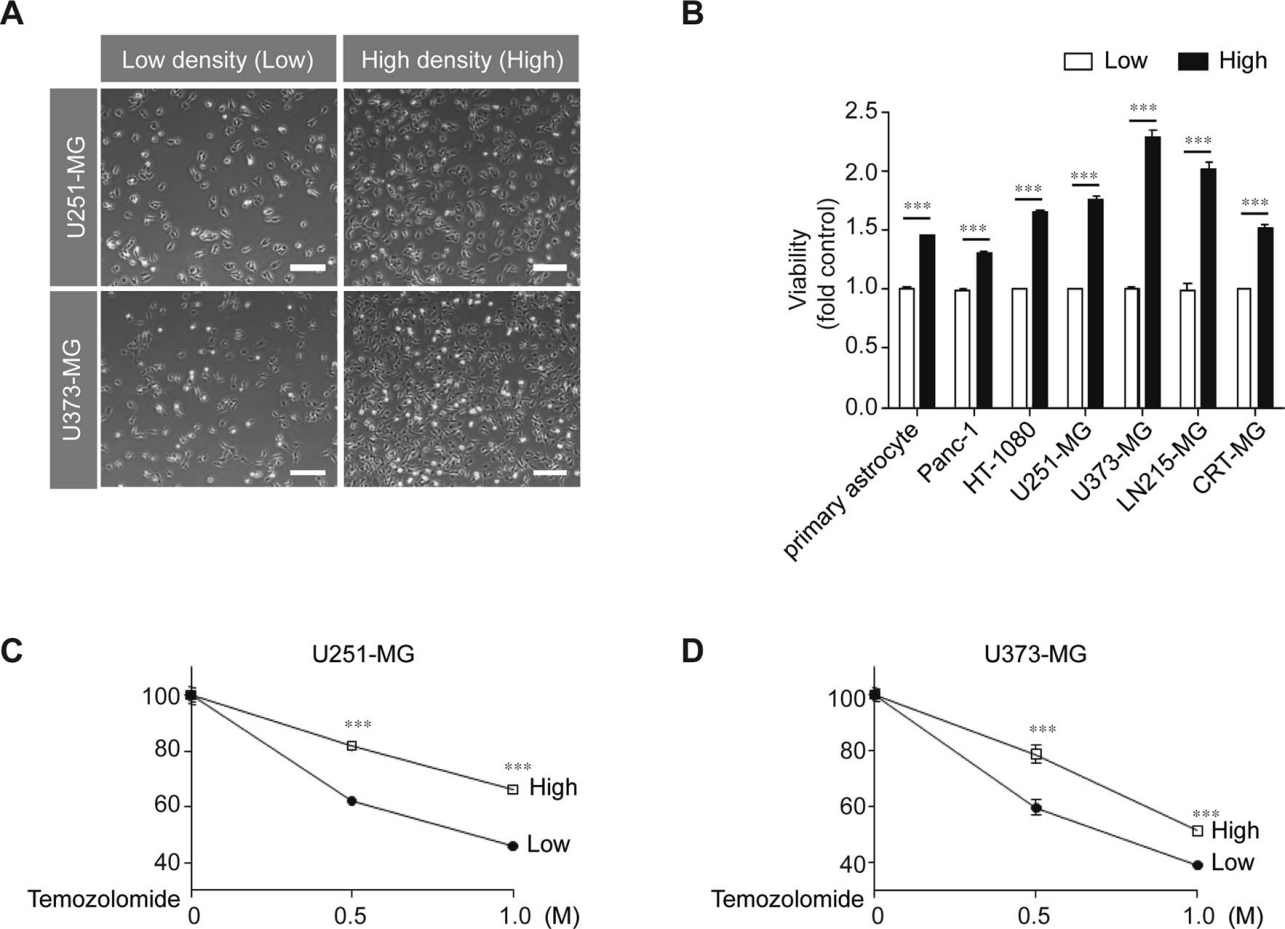
High-density culture conditions induce malignant features in various cancer cells. **A** U251-MG and U373-MG cells were cultured at low (9.09 × 10^3^ cells/cm^2^) or high density (2.38 × 10^4^ cells/cm^2^). Scale bar indicates 100 µm. **B** Primary astrocytes and several cancer cell lines were cultured at low or high density for 24 h, after which, cell viability was measured using a WST-1 assay (*n* = 3; Tukey’s post hoc test was used to detect significant differences in ANOVA, *p* < 0.001; asterisks indicate a significant difference compared to Low density and High density, ***p < 0.001). **C**, **D** U251-MG and U373-MG cells cultured at low or high density were treated with 0.5 M or 1 M TMZ for 24 h, after which, cell viability was measured by the WST-1 assay (*n* = 3; Tukey’s post hoc test was applied to detect significant differences in ANOVA, *p* < 0.0001; asterisks indicate a significant difference compared to 0% inhibition, ***p < 0.001).

Previously, we reported that HIF-1α could be induced in GBM cells without the use of a hypoxia chamber or hypoxia-inducing chemicals [25, 26]. Here, we wished to further determine whether cell-to-cell contact could be the cue for the induction of non-hypoxic HIF-1α expression in GBM cells outside of routine culturing conditions. To evaluate the effect of cell-to-cell contact on pro-tumorigenic features, various human cancer cell lines and human primary astrocytes were cultured to reach different confluencies after seeding the same number (5 × 10^5^) of cells in either 60 mm (high density, High) or 100 mm dishes (low density, Low) (Fig. 1A). As shown in Fig. 1B, primary astrocytes, pancreatic cancer cells (Panc-1), fibrosarcoma (HT-1080), and four GBM cell lines (U251-MG, U373-MG, LN215-MG, and CRT-MG) at high density showed approximately 20–100% higher viabilities than those at low density. We also found a correlation between cell-to-cell contact and the chemoresistance of malignant cancer cells. High-density cultures of U251-MG and U373-MG showed higher resistance to TMZ treatment than low-density cultures (Fig. 1C, D).

### Cell density-dependent pro-tumorigenic features are mediated by the Notch1 signaling pathway

**Fig. 2:**
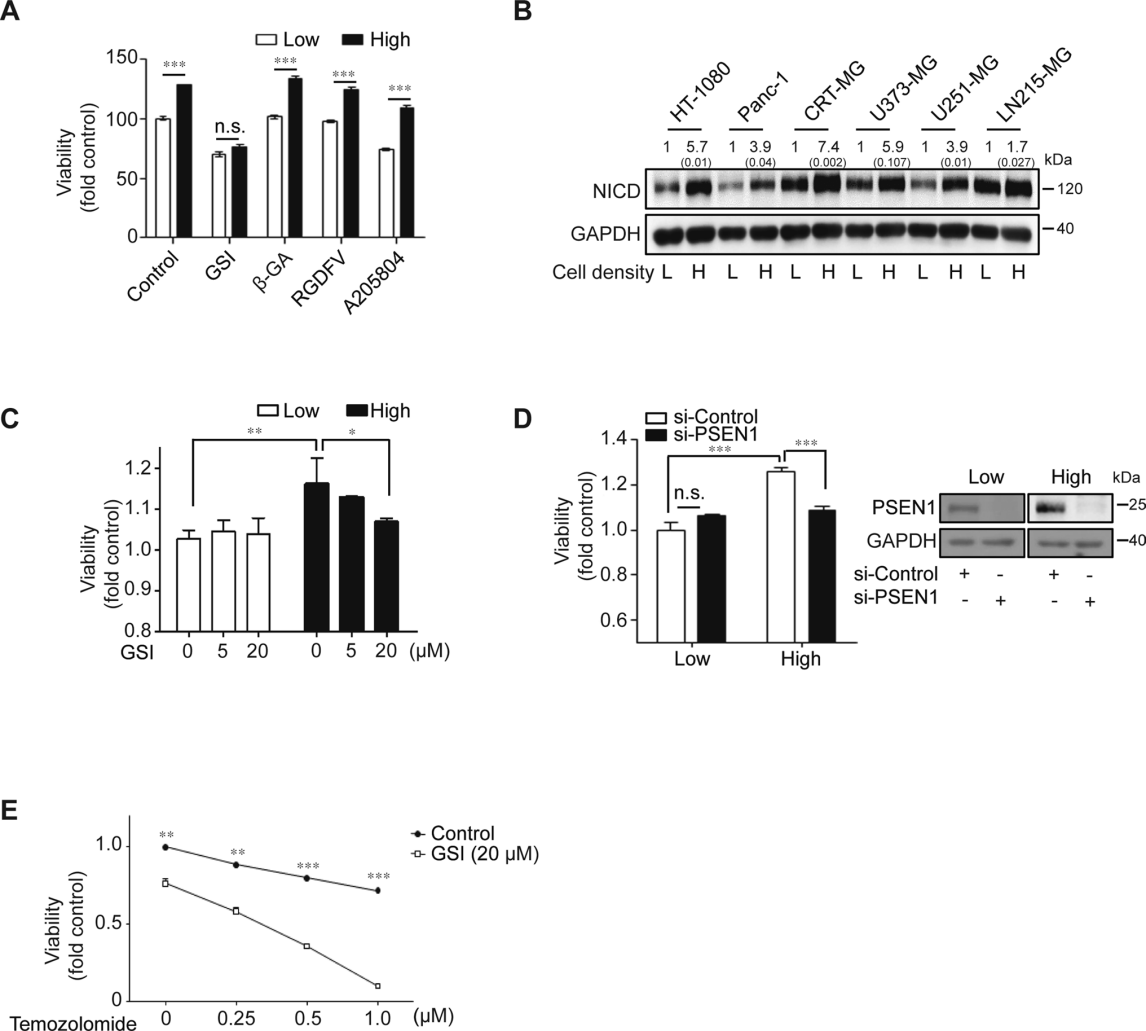
Ablating Notch1 signaling restrained density-dependent malignancy in various cancer cells. **A** U251-MG cells were treated with varying inhibitors associated with cell-to-cell interactions. GSI (50 μM), β-GA (50 μM), RGDFV (1 μM), or A205804 (30 μM) were administered for 24 h to low- or high-density cultured U251-MG cells, after which, cell viability was measured by the WST-1 assay (*n* = 3; Tukey’s post hoc test was used to detect significant differences in ANOVA, *p* < 0.001; asterisks indicate a significant difference compared to Low density and High density, ***p < 0.001). **B** The expression level of Notch intracellular domain (NICD) was measured by immunoblot assays in low- or high-density cultured cell lysates from HT-1080, Panc-1, CRT-MG, U373-MG, U251-MG, and LN215 cells. The relative pixel intensities were measured using ImageJ software. GAPDH was used as the loading control. The data are representative of three individual experiments. **C** U251-MG cells were treated with varying doses of GSI (0, 5, or 20 μM) for 24 h in low- or high-density cultured U251-MG cells, after which, cell viability was measured by the WST-1 assay (*n* = 3; Tukey’s post hoc test was applied to detect significant differences in ANOVA, *p* < 0.0001; asterisks indicate a significant difference compared to 0% inhibition, *p < 0.05, **p < 0.01). **D** Low- or high-density cultured U251-MG cells were transfected with control or PSEN1-targeting siRNA using Lipofectamine 2000™ reagent. After 48 h of siRNA transfection, cell viability was evaluated by the WST-1 assay (*n* = 3; Tukey’s post hoc test was used to detect significant differences in ANOVA, *p* < 0.001; asterisks indicate a significant difference compared to siControl and si-PSEN1, ***p < 0.001, *n.s.* non-significant). PSEN1 levels were significantly decreased in low- and high-density cultured U251-MG cells after si-PSEN1 transfections compared to si-Controls (Inset). **E** U251-MG cells were pre-treated with 20 μM of GSI. At 2 h post-treatment, the cells were incubated with or without varying doses of TMZ (0, 250, 500, and 1000 µM) for an additional 24 h, after which, cell viability was measured by the WST-1 assay (*n* = 3; Tukey’s post hoc test was applied to detect significant group effects in ANOVA, *p* < 0.0001; asterisks indicate a significant difference compared to 0% inhibition, *p < 0.05, **p < 0.01, ***p < 0.001)

To validate the mechanism of the density-dependent features of malignant cancer cells, various pharmaceutical inhibitors targeting inter-cellular contact (GSI against Notch1, β-GA against gap junction, RGDFV against integrin αvβ3/αvβ5, and A205804 against ICAM-1/E-selectin) were tested for the effects on U251-MG cells grown to high density. Ablation of the Notch1 signaling pathway by GSI specifically abrogated the high density-dependent viability of U251-MG cells, whereas other inhibitors had no effect on cell viability (Fig. 2A). High-density culturing resulted in the upregulation of NICD protein in HT-1080, Panc-1, CRT-MG, U373-MG, U251-MG, and LN215-MG cells (Fig. 2B). Blockade of Notch1 signaling by pretreatment with GSI strongly inhibited the enhanced viability of high-density U251-MG cells (Fig. 2C). The transfection of siRNA against PSEN1, encoding a key enzyme of the Notch1 signaling pathway, also decreased the enhanced viability of U251-MG cells, which was dependent upon cell density (Fig. 2D). Similar results were obtained using different si-PSEN1 oligonucleotides (data not shown). In addition, we validated the combined effect of GSI and TMZ treatment. GSI treatment (20 µM) induced a higher sensitivity of U251-MG cells to dose-dependent TMZ treatment compared to controls (Fig. 2E).

### High cell-to-cell contact regulates the expression levels of target transcription factors (TFs) in glioblastoma multiforme (GBM) cells

**Fig. 3:**
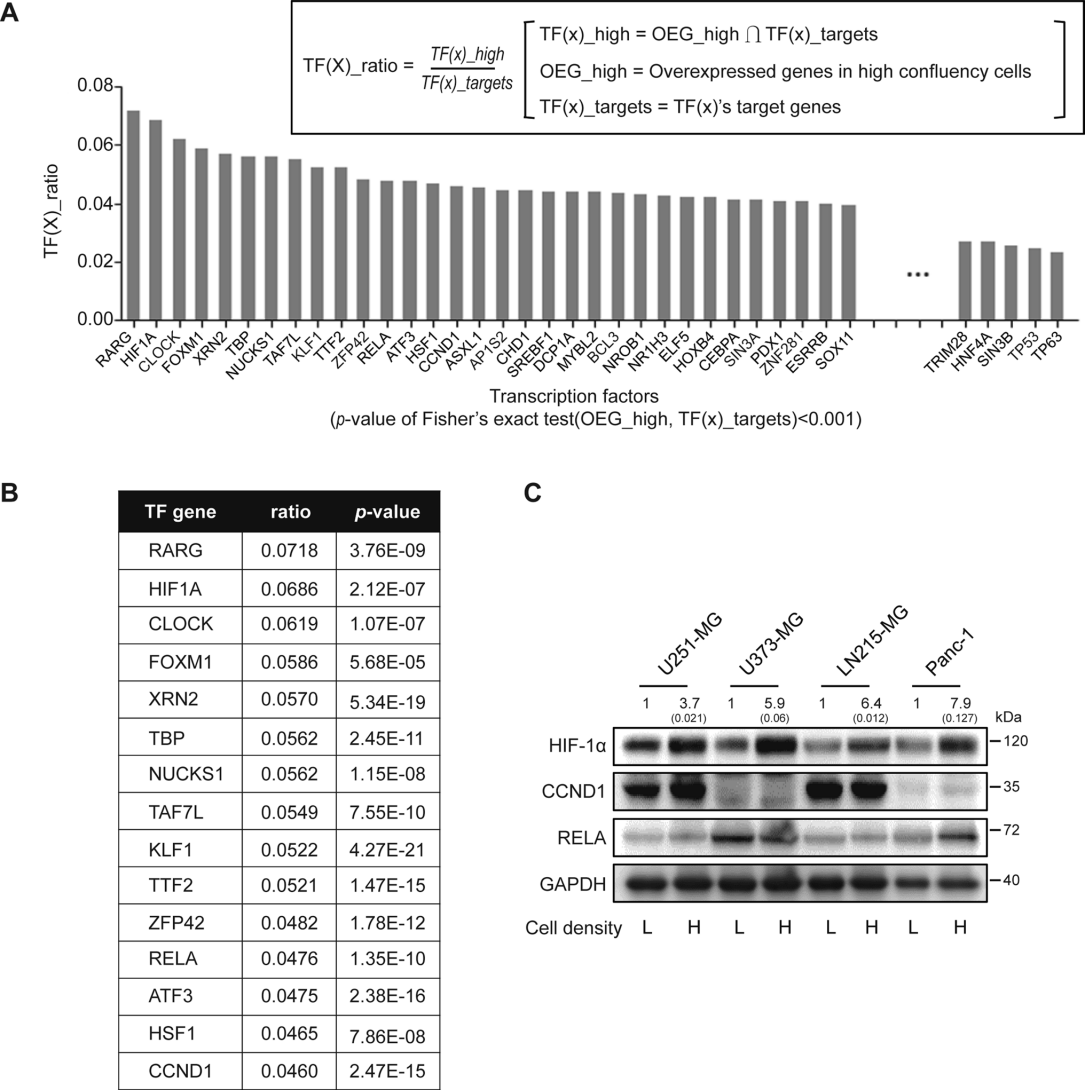
Expression of HIF-1α is associated with cell density-dependent culture conditions. **A** Total mRNA was extracted from low- or high-density cultured U251-MG cells and analyzed by next-generation sequencing (NGS). Genes highly expressed in high-density cultured cells are indicated as OEG-high, target gene sets of transcription factors (x) are indicated as TF(x)_targets, and the intersection of the OEG-high gene set and each TF(x)_target is designated as the TF(x)_ratio. Each TF(x)_ratio of 199 transcription factors was calculated. **B** The top 15 ranked transcription factors are displayed with *p* values. **C** Expression levels of HIF-1α encoded by HIF1A gene, cyclin D1 encoded by CCND1 gene, and p65 (NF-κB p65 subunit) encoded by RELA gene in low- or high-density cultured U251-MG, U373-MG, LN215-MG, and Panc-1 cells were evaluated by immunoblot assays (L indicates low-density culture; H indicates high-density culture). GAPDH was used as the loading control. Relative pixel intensities were measured using ImageJ software. The data are representative of three individual experiments

We performed next-generation sequencing (NGS) to investigate the differentially expressed genes in high-density U251-MG cells compared to low-density cultured cells. To examine TFs involved in the different gene expressions depending upon cell culture density, target TF genes overlapping with overexpressed genes in high-density cultures were analyzed. The TF(x)_ratio of each transcription factor for 199 TFs was calculated (Fig. 3A), and the rank of the TFs in accordance with their TF(x)_ratio was displayed up to the 15th rank (Fig. 3B). Among the top 15 ranked TFs, three (HIF-1α, CCND1, and p65) have been reported to be involved in the malignancy of GBM cells [27–29]. The expression level of HIF-1α was specifically elevated in high-density cultured GBM cells (U251-MG, U373-MG, and LN215-MG) and Panc-1 cells compared to the same cells at low density. There was no significant alteration in the expression of CCND1 in GBM and Panc-1 cells. However, the expression level of p65 was affected by cell density in Panc-1 cells, but not in GBM cells (Fig. 3C).

### Hypoxia-inducible factor-1α (HIF-1α) is regulated by high density-dependent Notch1 signaling

**Fig. 4:**
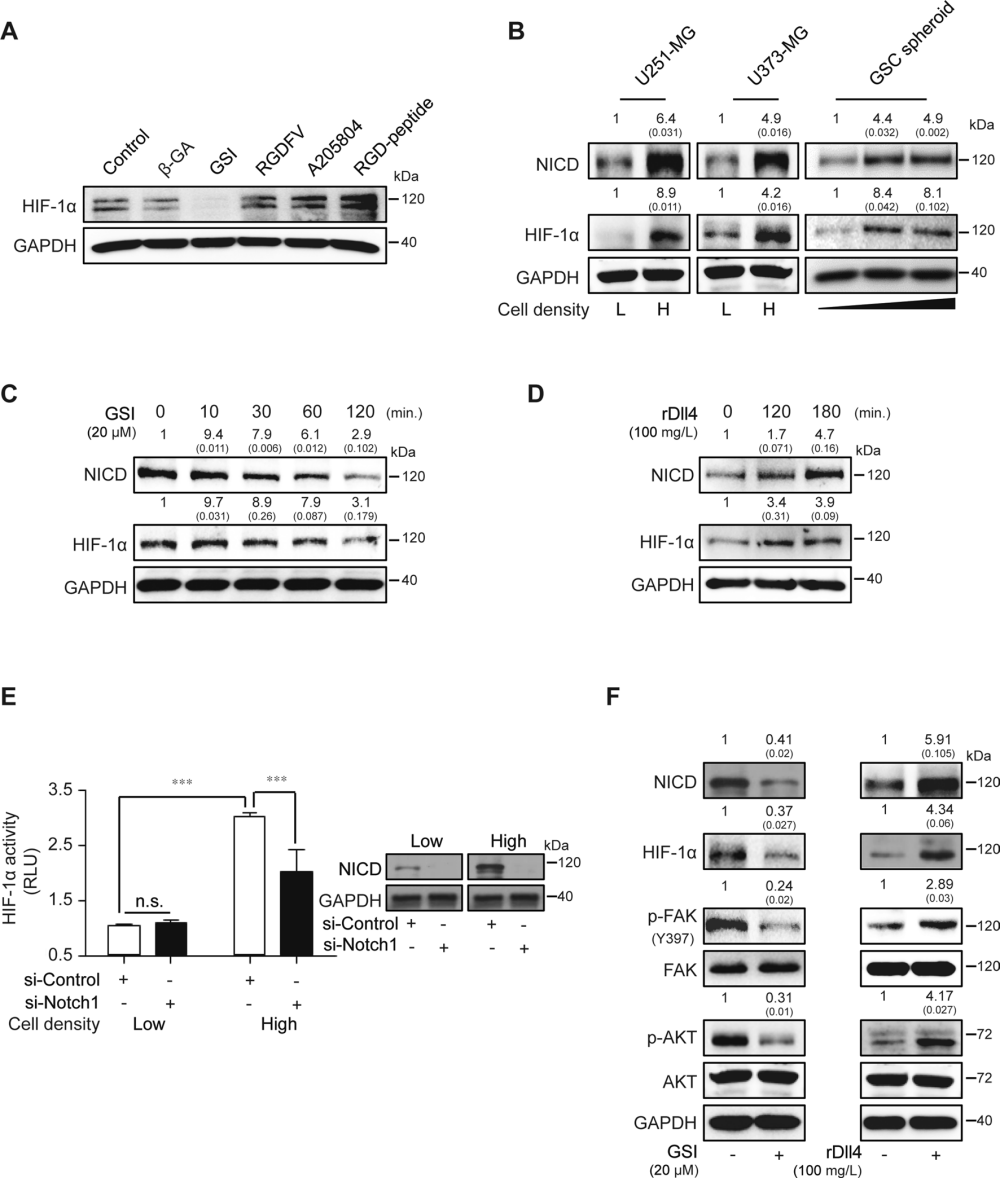
Cell density-dependent HIF-1α expression is modulated by Notch1 signaling regulation. **A** β-GA, GSI, RGDFV-peptide, A205804, or RGD-peptide were administered to high-density cultured U251-MG cells, and HIF-1α expression was evaluated by immunoblot assays. GAPDH was used as the loading control. The data are representative of three individual experiments. **B** NICD or HIF-1α expression was measured at different confluencies of U251-MG and U373-MG cells by immunoblot assays. Glioblastoma stem cells (GSC) were cultured for 1, 4, or 7 days to establish different sizes of spheroids, after which, NICD or HIF-1α were measured by immunoblot assays. GAPDH was used as the loading control. Relative pixel intensities were measured using ImageJ software. The data are representative of three individual experiments. **C**, **D** GSI (20 µM) or recombinant Dll4 (rDll4; 100 mg/L) were added to U251-MG cells in a time-dependent manner, and then NICD or HIF-1α levels were analyzed by immunoblot assays. GAPDH was used as the loading control. Relative pixel intensities were measured using ImageJ software. The data are representative of three individual experiments. **E** Low- or high-density cultured U251-MG cells were transfected with control or Notch1-targeting siRNA using Lipofectamine 2000™ reagent. After 48 h of siRNA transfection, HIF-1α activity was measured by the luciferase reporter assay (*n* = 3; Tukey’s post hoc test was applied to detect significant group effects in ANOVA, *p* < 0.0001; asterisks indicate a significant difference compared to 0% inhibition, ***p < 0.001, n.s.: non-significant). NICD levels were significantly decreased in low and high-density cultured U251-MG cells after si-Notch1 transfections compared to si-Controls (Inset). **F** Low- or high-density cultured U251-MG cells were maintained for 24 h, after which, the expression levels of NICD, HIF-1α, p-FAK(Y397), FAK, p-AKT, AKT, or GAPDH were evaluated by immunoblot assays. Relative pixel intensities were measured using ImageJ software. The data are representative of three individual experiments

To investigate the correlation between multiple cell surface proteins and non-hypoxic HIF-1α expression in a state of high cell-to-cell contact, pharmaceutical inhibitors including β-GA, GSI, RGDFV-peptide, A205804, and RGD-peptide were used to treat high-density U251-MG cells. Treatment with GSI specifically diminished the expression of non-hypoxic HIF-1α in high-density U251-MG cells, whereas other inhibitors had no effect on HIF-1α expression (Fig. 4A). NICD release and HIF-1α expression were enhanced in high-density U251-MG and U373-MG cells compared to low-density cells. Glioblastoma stem cell (GSC) spheroids of different sizes were also investigated (Fig. 4B). Non-hypoxic HIF-1α induced by enhanced cell density was shown to functionally regulate the production of vascular endothelial growth factor (VEGF)-A in U251-MG cells (Supplementary Fig. 1). We further examined whether non-hypoxic HIF-1α expression was dependent upon Notch1 signaling. HIF-1α expression in U251-MG cells was decreased by GSI treatment during stimulation by exogenous treatment with recombinant Dll4, a ligand of the Notch1 receptor (Fig. 4C, D). HIF-1α activity was also increased in high-density cultures compared to low density, an effect which was inhibited by si-Notch1 transfection in U251-MG cells (Fig. 4E). Similar results were obtained using different si-Notch1 oligonucleotides (data not shown). Previously, we demonstrated that the FAK and AKT signaling pathways played critical roles in aggravating GBM both in vitro and in vivo [26]. Based on this, we investigated intracellular signaling following treatment with GSI or recombinant Dll4 in high-density U251-MG cells. As shown Fig. 4F, treatment with these reagents regulated the phosphorylation of FAK or AKT compared to non-treated U251-MG cells.

### NICD release restrains the hydroxylation of hypoxia-inducible factor-1α (HIF-1α) through reciprocal interaction

**Fig. 5:**
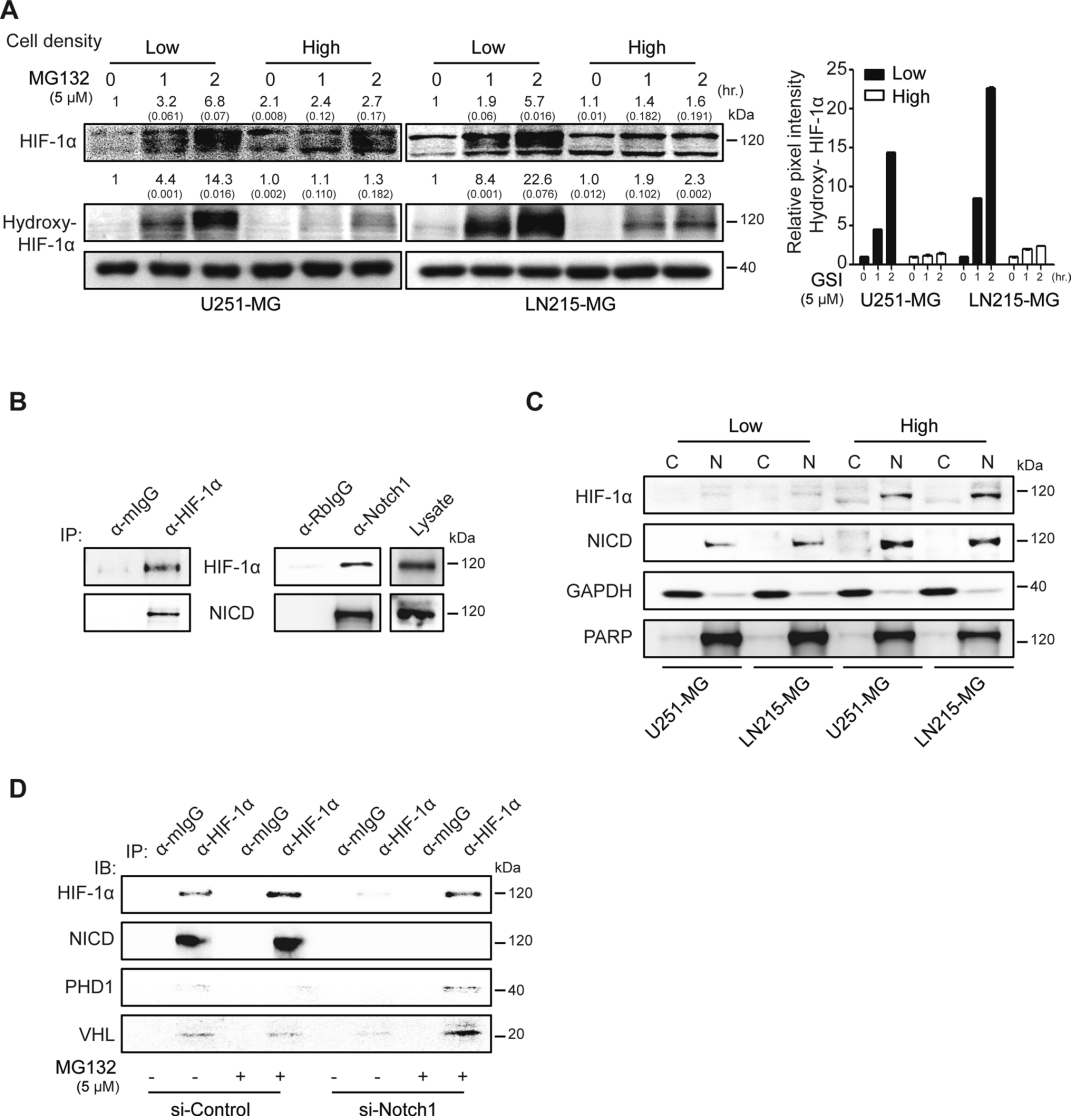
Interaction between NICD and HIF-1α prevents HIF-1α hydroxylation and degradation. **A** Low- or high-density cultured U251-MG and LN215-MG cells were treated with 5 μM of the proteasome inhibitor MG132 for different times (0, 1, or 2 h). In each cell lysate, HIF-1α or hydroxylated HIF-1α (hydroxy-HIF-1α) was evaluated by immunoblot assays. GAPDH was used as the loading control. Relative pixel intensities were measured using ImageJ software. The data are representative of three individual experiments. **B** High-density cultured U251-MG cell lysates were bound to anti-HIF-1α or normal IgG antibodies, followed by protein G agarose addition. The immunoprecipitated proteins were then detected by HIF-1α or NICD antibodies in immunoblot assays. Non-bound lysate was used for the loading control. **C** Low- or high-density cultured U251-MG and LN215-MG cells were separated into cytosolic and nuclear fractions, and HIF-1α or NICD levels were analyzed by immunoblot assays. GAPDH or PARP was used as the loading controls for the cytosolic and nuclear fractions, respectively. Relative pixel intensities were measured using ImageJ software. The data are representative of three individual experiments. **D** U251-MG cells were transfected with siControl or si-Notch1 for 48 h and treated with MG132 for 2 h before preparing cell lysates. Lysates immunoprecipitated with anti-HIF-1α or normal mouse IgG antibodies were immunoblotted using HIF-1α, NICD, PHD, or VHL antibodies. The data are representative of three individual experiments

To validate the functional role of NICD release in non-hypoxic HIF-1α expression, we evaluated the hydroxylation of HIF-1α in U251-MG and LN215-MG cells in high- or low-density cultures after exposure to MG132, a proteasome inhibitor. Hydroxylated HIF-1α was specifically accumulated in a time-dependent manner after MG132 treatment in low-density culture conditions. However, hydroxylation was strongly decreased at high density in U251-MG and U373-MG cells after identical treatment with MG132 (Fig. 5A). To further investigate the physical interaction between NICD and HIF-1α in non-hypoxic conditions, we performed immunoprecipitation assays using the lysates of densely cultured U251-MG cells. HIF-1α and NICD reciprocally interacted in U251-MG cells (Fig. 5B), and complexes were mainly accumulated in the nuclei (Fig. 5C). To reveal the role of NICD in non-hypoxic HIF-1α stability, MG132 was used on scrambled-siRNA or si-Notch1-transfected high-density U251-MG cells. HIF-1α interacted with NICD, but not with PHD or VHL, in control-siRNA transfected U251-MG cells. However, silencing Notch1 expression specifically resulted in the co-precipitation of PHD or VHL with HIF-1α under exposure to MG132 (Fig. 5D).

### In vivo effects of hypoxia-inducible factor-1α (HIF-1α) blockade in human glioblastoma multiforme (GBM) cells

**Fig. 6:**
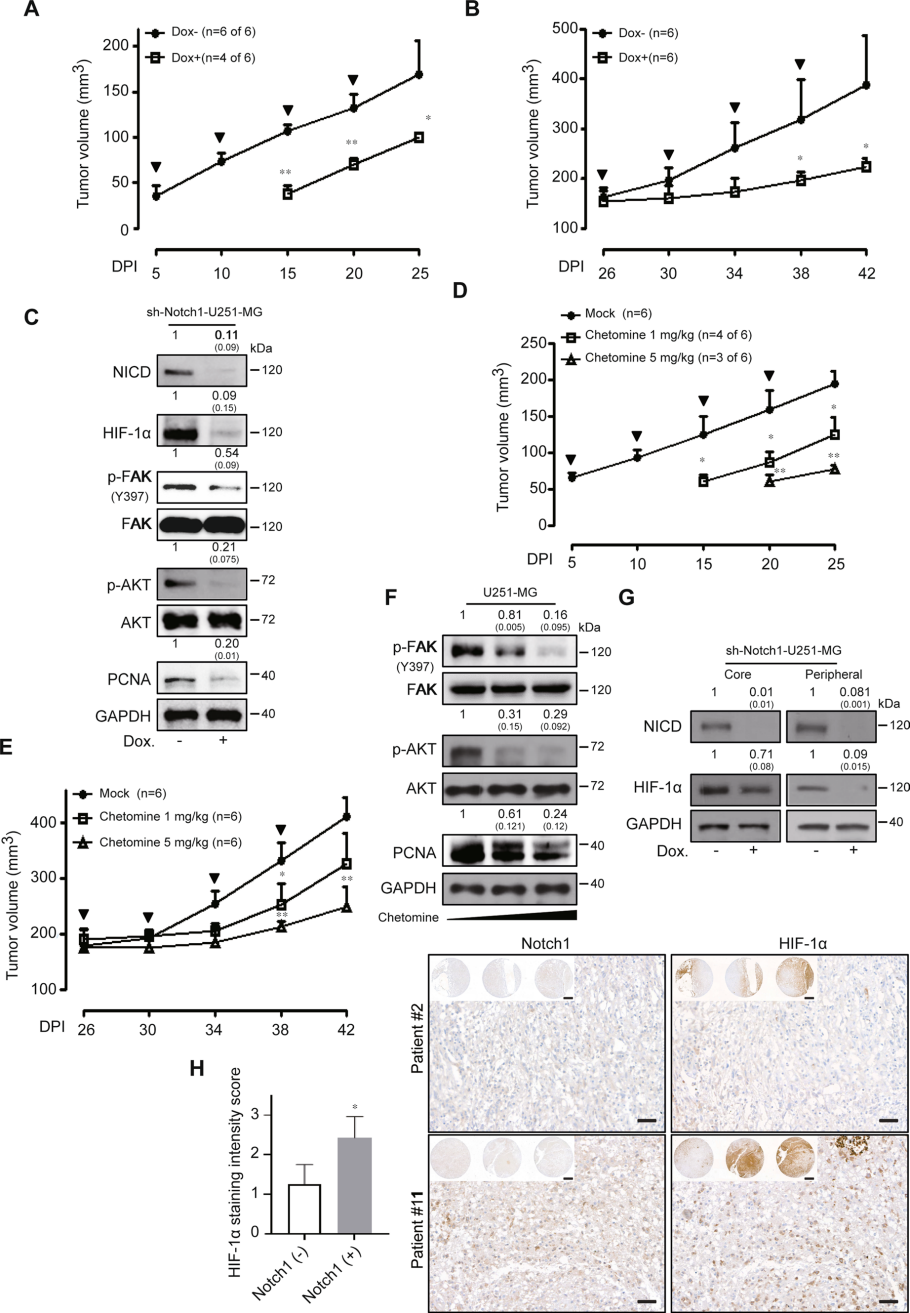
Blockade of abnormal HIF-1α induces in vivo anti-cancer effects in GBM cells. **A**, **B** sh-Notch1-Dox^−^ (*n* = 6) and sh-Notch1-Dox^+^ (*n* = 6) U251-MG xenografted tumors were measured for 25 or 42 days. Bold arrows indicate the time of doxycycline treatment (Tukey’s post hoc test was used to detect significant differences in ANOVA, *p* < 0.0001; asterisks indicate a significant difference compared to sh-control and sh-Notch1, *p < 0.05, **p < 0.01, *n.s.* non-significant). **C** Western blot analysis of sh-Notch1-Dox^−^ and sh-Notch1-Dox^+^ tumor lysates using anti-Notch1, HIF-1α, p-FAK, FAK, p-AKT, AKT, and PCNA antibodies. GAPDH was used as the loading control. Relative pixel intensities were measured using ImageJ software. The data are representative of three individual experiments. **D**, **E** The effects of chetomin (0, 1, or 5 mg/kg) on tumor size in U251-MG xenograft models were analyzed after 25 and 42 days. The bold arrows indicate the time of chetomin injection (Tukey’s post hoc test has been used to determine significant group effects in ANOVA, *P* < 0.0001; asterisks indicate a significant difference between the control group and chetomin injection group, *P < 0.05). **F** Western blot analysis of 0, 1, or 5 mg/kg chetomin injection tumor lysates using anti-p-FAK, FAK, p-AKT, AKT, and PCNA antibodies. GAPDH was used as the loading control. Relative pixel intensities were measured using ImageJ software. The data are representative of three individual experiments. **G** Regional lysates (peripheral or core region) from sh-Notch1-Dox^−^ and sh-Notch1-Dox^+^ tumor samples were analyzed by immunoblotting for HIF-1α or NICD expression. GAPDH was used as the loading control. Relative pixel intensities were measured using ImageJ software. The data are representative of three individual experiments. Peripheral region refers to the outer 75% of the tumor sample sections. **H** Left, comparison of HIF-1α staining intensity scores between the Notch1-negative staining group (*n* = 4) and the Notch1-positive staining group (*n* = 7) among positively expressing HIF-1α patients. Right, representative images of GBM patient tissue microarray analysis (scale ba*r* = 50 μm; inset image scale bar = 500 μm)

We constructed a doxycycline-inducible sh-Notch1 U251-MG cell line and then investigated the effect of specifically targeting non-hypoxic HIF-1α in vivo. First, we checked the efficacy of NICD knock-down to affect non-hypoxic HIF-1α levels in vitro. Treatment with doxycycline induced a significant reduction in NICD accompanied by the inhibition of HIF-1α (Supplementary Fig. 2A). The cell viability of sh-Notch1 U251-MG cells was also suppressed by treatment with doxycycline compared to control cells (Supplementary Fig. 2B). The anti-growth effect of Notch1 and HIF-1α blockade was observed in doxycycline-inducible sh-Notch1 U251-MG xenograft models in different ways. Mice transplanted with sh-Notch1 U251-MG cells were fed doxycycline from the first day to the 25th day every 5 days. At 25 days after transplantation, the control tumors grew to an average size of 169.17 ± 36.7 mm^3^, whereas the shNotch-inducible tumors had an average size of 100.34 ± 4.33 mm^3^. Tumor appearance in sh-Notch1 U251-MG xenografted mice was delayed 10 days compared to the tumors in control mice. Moreover, four of six doxycycline-fed mice xenografted with sh-Notch1 U251-MG cells developed tumors compared to six of six xenografted mice not fed doxycycline (Fig. 6A). Mice transplanted with sh-Notch1 U251-MG cells were also fed doxycycline when the tumors reached an average size of approximately 170 mm^3^. The control tumors grew to an average size of 387.53 ± 100 mm^3^ 42 days after transplantation, whereas the shRNA-inducible tumors remained at an average of 223.63 ± 15.8 mm^3^ at 42 days (Fig. 6B). No weight loss was detected in the Dox^+^ or Dox^−^ xenograft models (data not shown). Subsequently, we performed a Western blot analysis using the lysates of untreated or treated doxycycline sh-Notch1 xenograft tumors to investigate in vivo intracellular signaling and the mechanisms of the anti-tumor effect. NICD, HIF-1α, phospho-FAK, and phospho-AKT were strongly reduced in doxycycline-treated sh-Notch1 U251-MG xenografts compared to those in the untreated sh-Notch1 U251-MG xenograft models. We also investigated the levels of proliferating cell nuclear antigen (PCNA) in the lysates of untreated and treated doxycycline sh-Notch1 xenograft tumors and found that PCNA was diminished following NICD and HIF-1α blockade in sh-Notch1 U251-MG xenografts (Fig. 6C). We further investigated the anti-tumor effect of chetomin, an inhibitor of the HIF pathway, in U251-MG xenograft models using the same regimen as that used for the doxycycline sh-Notch1 xenograft models. As expected, similar in vivo anti-tumor results were obtained (Fig. 6D, E). The intracellular signaling involved was analyzed by Western blots (Fig. 6F). To verify the in vivo expression of non-hypoxic HIF-1α in GBM cells, we subsequently performed Western blot analysis using core or peripheral regional lysates of the tumors. In the central regions, HIF-1α was highly expressed in Dox^−^ tumor lysates, whereas it was weakly decreased in the setting of reduced Notch1. In contrast, in the peripheral regions, the level of HIF-1α protein was clearly lower in Dox^−^ tumor lysates than in the core. However, it was notably reduced by the blockade of Notch1 expression (Fig. 6G). To further verify the clinical relevance of the correlation between Notch1 and HIF-1α expression, we performed a tissue microarray analysis of GBM patient samples. Of a total of 19 GBM patients enrolled, HIF-1α was expressed in the samples from 11 patients but not observed in the samples from eight patients. Because sample bias may exist between hypoxic or non-hypoxic regions during surgical specimen acquisition, tissue microarray analysis was performed only on the HIF-1α-expressing samples. Among the 11 samples expressing HIF-1α, Notch1 was not expressed in four (36.4%), but was expressed in seven of the patient samples (63.6%). From an analysis of the expression level of HIF-1α (with or without Notch1 expression), which was based on the staining intensity score judged by an expert pathologist who analyzed the samples in a blinded fashion, the expression level of HIF-1α in GBM patient samples was positively correlated with Notch1 expression status (Notch1-negative, 1.25 ± 0.50; Notch1-positive, 2.43 ± 0.53; *p* < 0.05) (Fig. 6H).

**Fig. 7:**
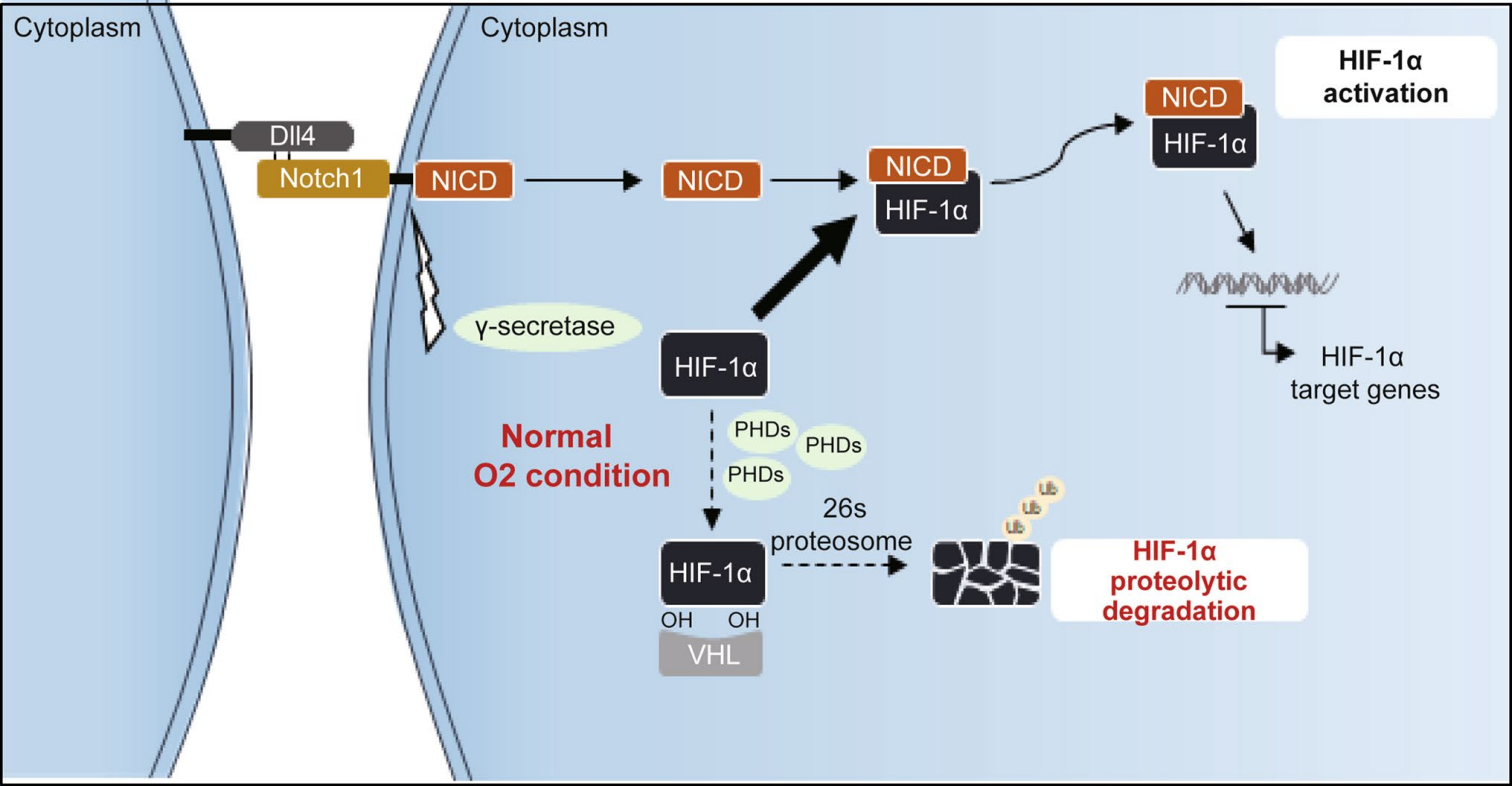
Graphical summary of non-hypoxic HIF-1α stabilization in GBM cells

Together, our data indicate that functional HIF-1α was stabilized from proteasomal degradation by interactions with NICD in human GBM cells, and this interaction was critical for the malignancy of GBM cells, even in a non-hypoxic setting (Fig. 7).

## Discussion

The results of the present study revealed correlations between cell-to-cell contact induced by cell density and malignant features of GBM such as viability and sensitivity against TMZ. The blockade of Notch1 signaling by γ-secretase inhibitor treatment or siRNA transfection inhibited the high cell density-induced malignant features of GBM cells. High cell density increased various transcription factors including HIF-1α and specifically induced HIF-1α protein expression even in normal culture conditions compared to other transcription factors such as CCND1 and p65. Of interest, atypical non-hypoxic HIF-1α was preserved in its functional expression by escaping proteolytic degradation through interacting with the Notch1 intracellular domain. We also evaluated the in vivo anti-cancer effects following ablation of the HIF-1α signaling pathway using a doxycycline-inducible Notch1 RNA-interfering system or by treating cells with chetomin and validated the correlation between HIF-1α and Notch1 expression using clinical tissues from GBM patients.

Glioblastoma multiforme (GBM) is the most aggressive malignant primary brain tumor in adults [30]. Although surgical resection with adjuvant therapies has been refined constantly over time, the prognosis of patients with GBM remains poor [31]. GBM has a remarkable tendency to exhibit out-of-control progression characterized by widespread invasion throughout the brain parenchyma with unclear tumor margins. This propensity is a major cause of the approximately 100% recurrence rate, resulting in an average life expectancy of only 12–14 months [32, 33]. Understanding the mechanisms underlying GBM malignancy and developing therapeutic reagents or targeted strategies against GBM is critical to addressing the poor clinical outcomes.

Along with various extracellular cues such as chemical factors, autocrine/paracrine growth factors, and oxygen availability, juxtacrine signaling is a key signaling pathway induced by cell-to-cell contact, which regulates several signaling pathways including cadherins, integrins, and Notch1 signaling [16, 17]. GBM has a limited area to grow due to cranial bones, resulting in a serried or competitive survival environment. Unlike functional normal cells, tumor cells can escape cell-to-cell contact-dependent self-inhibitory effects and grow over each other indefinitely in an immortal manner [34, 35]. Such limitless growth of tumor cells is one of the major hallmarks of cancer [36], resulting in the invasion of surrounding tissues and eventually, metastasis. Consistent with previous studies, our results revealed that various tumor cells including GBM cells at high cell density showed significantly enhanced growth and chemoresistance to TMZ, strongly suggesting that cell-to-cell contact is a critical cue that regulates the viability and chemoresistance of GBM cells.

Candace et al. reported the cytotoxic effects of the combination of γ-secretase inhibitor (GSI) and TMZ on GBM cells ex vivo and in vivo [37]. The present study validated an interesting function of the blockade of Notch1 signaling using γ-secretase inhibitor (GSI) or transfection with si-PSEN1, in that the blockade specifically restrained high cell density-induced viability compared to other cell surface molecule inhibitors, including gap junctions, integrins αvβ3/αvβ5, and ICAM-1/E-selectin inhibitors. In addition, ablating Notch1 signaling by treatment with GSI showed a synergistic anti-viability effect with dose-dependent TMZ treatment, indicating that the Notch1 signaling pathway might be responsible for the growth and chemoresistance of GBM cells induced by cell-to-cell contact. Thus, we cannot ignore the potential synergistic cytotoxic effect of Notch1 blockade with other chemotherapeutic drugs against GBM.

The role of HIF in tumor biology has received a great deal of attention recently. It has been extensively investigated for its underlying functions and the signaling that occurs specifically in hypoxic conditions [38, 39]. In malignant tumors, HIF-1α is associated with oncogenic angiogenesis, proliferation, invasion, metastasis, and resistance to apoptosis [13, 40]. Previously, we reported that HIF-1α was upregulated in GBM cells even under normal culture conditions [25, 26]. The results of the present study shed further light on the underlying mechanism of non-hypoxic HIF-1α expression for the first time. We demonstrated that HIF-1α mRNA expression was upregulated by high cell confluency (at about 20% oxygen concentration) [41] through NGS analysis and further proved that HIF-1α protein levels were increased at high cell density. To the best of our knowledge, this is the first report to show that cell-to-cell contact might be a critical stimulus regulating HIF-1α expression at both the mRNA and protein levels. With the above data, we were able to address a fundamental question related to cell density phenomena and more importantly, how cell-to-cell contact could render functional malignancy to GBM cells. Previous studies have reported on the relationship between NICD and HIF-1α in GBM. In a hypoxic microenvironment, activated HIF-1α could maintain GBM stem cells by stimulating Notch1 signaling and promoting the migration and invasion of choriocarcinoma cells [42, 43]. Consistent with, and expanding on our earlier work, these results unravel the mechanisms underlying non-hypoxic HIF-1α expression via the Notch1 signaling pathway in GBM cells by cell-to-cell contact even under non-hypoxic condition.

The expression of HIF-1α was specifically diminished by silencing Notch1 signaling with GSI treatment, whereas inhibiting gap junctions, integrins, or ICAM-1/E-selectin in GBM cells had no effect. In addition, HIF-1α and NICD were enhanced by cell-to-cell contact in GBM cells and GBM stem cells. As the number of days in culture increased, the size of the GSC spheroids increased, indicating that more cells contacted each other. Notably, HIF-1α protein was regulated by treatment with GSI or recombinant Dll4 at a relatively short exposure time of 2 h, implying that modulating HIF-1α protein might be involved as an escape pathway from proteasomal degradation, which is the routine fate of HIF-1α in normoxic conditions [13]. We validated that the cell-to-cell contact-dependent interaction of NICD with HIF-1α allowed competitive evasion from the action of VHL or PHD to lyse HIF-1α in GBM cells, even in non-hypoxic conditions. However, the specific binding site of HIF-1α with NICD remains unclear. Cobalt chloride is known to stabilize HIF-1α in a hypoxia-independent manner by inhibiting prolyl hydroxylase [44, 45]. However, unlike cobalt chloride, HIF-1α and NICD demonstrated a physical interaction, which prevented the proteasomal degradation process. For a more detailed view of this signaling, the binding site of HIF-1α with NICD should be further investigated.

The freedom from contact inhibition exhibited by tumor cells might be an undiscovered cue for their malignant processes according to previous reports involving pericellular hypoxia [46–48]. Due to this gain of function, the unruly proliferation of tumor cells enables them to establish a highly dense tumor microenvironment, which provides a high cell-to-cell contact ratio even when cancer sizes are small in the early stages. Evolutionary courses with heterogeneous subpopulations of tumor render the adaptation to their microenvironment and survival against extracellular cues [49]. To validate HIF-1α as a therapeutic target for a small cell population or an early stage of cancer progression prior to the creation of a hypoxic core, we established in vivo models using a doxycycline-inducible sh-Notch1 U251-MG cell line and chetomin. Silencing Notch1 signaling or HIF-1α inhibition showed a notable anti-growth effect in early treatment with doxycycline or chetomin, indicating that HIF-1α might be a promising therapeutic target even in an early stage of tumor progression. HIF-1α is tightly governed by the tumor microenvironment. However, hypoxia caused by low O_2_ concentrations can critically shift this balance to aggressive growth, employing HIF-1α as a transcription factor [13–15]. Consistent with this, the present in vivo data indicated that ablating HIF-1α by silencing Notch1 as a guard-molecule or by treating with chetomin, a HIF pathway inhibitor, induced significant anti-tumor effects in GBM xenograft models. Additionally, our mouse data extended the knowledge concerning the fate of HIF-1α in the tumor microenvironment. HIF-1α expression was diminished not only in the central core region but also in the peripheral regions where blood perfusion is higher and was accompanied by a reduction in Notch1 expression, implying that functional HIF-1α was maintained by associating with Notch1 even in the non-hypoxic setting. In addition, non-hypoxic-HIF-1α might be the critical player to hatch from the occult dormancy of GBM after surgical removal or tumor treatment [50–52]. The correlation between preserving non-hypoxic-HIF-1α and escaping dormancy in GBM remains critical issue. To embody the scenario, further systemic device or techniques absolutely should be established against the limitation of routine cultured conditions [41]. Importantly, despite the limitations arising from the small number of patients, the analysis of patient samples using TMA provided enhanced evidence for the correlation between Notch1 and HIF-1α expression. Further study using a large number of patient tissues location defining fine information of tissue locations should be performed. Based on the previous reports associated with T2/FAIR on magnetic resonance imaging for detecting the GBM dissemination or invasion [53, 54], application of the technique to correlation between HIF-1α and Notch1 expression in non-hypoxic environment may lead to more detailed validation results.

## Conclusions

Collectively, our results revealed that cell-to-cell contact could sustain the function of HIF-1α through its association with the Notch1 signaling pathway even in a non-hypoxic setting. In addition, non-hypoxic HIF-1α expression could be present normally and generated by different extracellular regulatory pathways. Moreover, the present report emphasizes the importance of atypical HIF-1α as a potential stimulus for GBM progression or recurrence even in a non-hypoxic environment. Taken together, our results indicate that atypical HIF-1α might be a promising therapeutic target against GBM even in a non-hypoxic microenvironment.

## Supplementary Information

Below is the link to the electronic supplementary material.

Supplementary file1 (DOCX 54 KB)

## Data Availability

Data will be made available on reasonable request.

## Abbreviations

HIF-1α: Hypoxia-inducible factor-1α
GBM: Glioblastoma multiforme
TMZ: Temozolomide
siRNA: Small interfering RNA
IP: Immunoprecipitation
IHC: Immunohistochemistry
