# Supplementary material for: Atypical induction of HIF-1α expression by pericellular Notch1 signaling suffices for the malignancy of glioblastoma multiforme cells
Source: Cell Mol Life Sci. 2022 Oct 2;79(10):537. doi: 10.1007/s00018-022-04529-2 (PMC9527190; doi:10.1007/s00018-022-04529-2)
Supplement: Supplementary file 1 — Supplementary file1 (DOCX 54 KB) [file 18_2022_4529_MOESM1_ESM.docx]

**Atypical induction of HIF-1α expression by pericellular Notch1 signaling suffices for the malignancy of glioblastoma multiforme cells**

**Authors and Affiliations**

Jungwhoi Lee^1^**^ǂ^***, Eunsoo Kim^2^**^ǂ^**, Kyuha Chong^3^**^ǂ^**, Seung-Wook Ryu^2^, Chungyeul Kim^4^, Kyungsun Choi^2^, Jae-Hoon Kim^1^, and Chulhee Choi^2,5^*

^1^Department of Applied Life Science, SARI, Jeju National University, Jeju-do 63243, Republic of Korea

^2^ILIAS Biologics Inc., 40-20, Techno 6-50, Yuseong-gu, Daejeon 34014, Republic of Korea

^3^Department of Neurosurgery, Korea University Guro Hospital, Korea University Medicine, Korea University College of Medicine, 148 Gurodong-ro, Guro-gu, Seoul, 08308, Republic of Korea

^4^Department of Pathology, Korea University Guro Hospital, Korea University Medicine, Korea University College of Medicine, 148 Gurodong-ro, Guro-gu, Seoul, 08308, Republic of Korea

^5^Department of Bio and Brain Engineering, KAIST, Daejeon 34141, Republic of Korea

**Supplementary Figure 1.** **Expression of VEGF-A is associated with cell density-dependent culture condition.**

An ELISA for quantity of VEGF-A was performed using cultured supernatants from low or high density U251-MG cells (The *p* value was determined using Student’s *t* test, ***p* < 0.01).

Supplementary Figure 2. Blockade of Notch1 is associated with regulating HIF-1α expression.

**(A)** Western blot analysis of sh-Notch1-Dox+ U251-MG cell lysates using anti-Notch1 and -HIF-1α antibodies. GAPDH was used as loading control. Relative pixel intensities were measured using ImageJ software. The data are representative of three individual experiments. **(B)** Doxycycline (Dox.; 0, 10, or 15 mg/L) was added to U251-MG-sh-Notch1 cells for 72h, after which cell viability was measured using a WST-1 assay (*n* = 3; Tukey’s *post-hoc* test was used to detect significant differences in ANOVA, *p* < 0.001; asterisks indicate significant difference compared to 0% inhibition, **p* < 0.05, ****p* < 0.001).
